# Attitudes Toward Seeking Professional Psychological Help: Factor Structure and Socio-Demographic Predictors

**DOI:** 10.3389/fpsyg.2016.00547

**Published:** 2016-04-25

**Authors:** Louisa Picco, Edimanysah Abdin, Siow Ann Chong, Shirlene Pang, Saleha Shafie, Boon Yiang Chua, Janhavi A. Vaingankar, Lue Ping Ong, Jenny Tay, Mythily Subramaniam

**Affiliations:** ^1^Research Division, Institute of Mental HealthSingapore, Singapore; ^2^Department of Psychology, Institute of Mental HealthSingapore, Singapore

**Keywords:** attitudes, psychological help-seeking, mental illness, Singapore

## Abstract

Attitudes toward seeking professional psychological help (ATSPPH) are complex. Help seeking preferences are influenced by various attitudinal and socio-demographic factors and can often result in unmet needs, treatment gaps, and delays in help-seeking. The aims of the current study were to explore the factor structure of the ATSPPH short form (-SF) scale and determine whether any significant socio-demographic differences exist in terms of help-seeking attitudes. Data were extracted from a population-based survey conducted among Singapore residents aged 18–65 years. Respondents provided socio-demographic information and were administered the ATSPPH-SF. Weighted mean and standard error of the mean were calculated for continuous variables, and frequencies and percentages for categorical variables. Confirmatory factor analysis and exploratory factor analysis were performed to establish the validity of the factor structure of the ATSPPH-SF scale. Multivariable linear regressions were conducted to examine predictors of each of the ATSPPH-SF factors. The factor analysis revealed that the ATSPPH-SF formed three distinct dimensions: “Openness to seeking professional help,” “Value in seeking professional help,” and “Preference to cope on one's own.” Multiple linear regression analyses showed that age, ethnicity, marital status, education, and income were significantly associated with the ATSPPH-SF factors. Population subgroups that were less open to or saw less value in seeking psychological help should be targeted via culturally appropriate education campaigns and tailored and supportive interventions.

## Introduction

There has been a growing interest in people's attitudes toward seeking psychological help. While recent research has shown an increase in the number of people seeking help from psychological services, there is still a significant number who choose not to seek help for mental health problems. This underutilization is often related to stigma (Jorm et al., 2007; Gulliver et al., 2010), reluctance to disclose a diagnosis (Hinson and Swanson, 1993) and anticipated costs (Vogel and Wester, 2003). In addition, attitudinal barriers such as choosing to handle the problem on one's own (Rickwood et al., 2007; Gulliver et al., 2010; Chong et al., 2012b; Wilson and Deane, 2012) and thinking the problem will go away (Thompson et al., 2004; Sareen et al., 2007) further contribute to underutilization of mental health services.

Other important components which may influence help-seeking for mental health problems include the perceived helpfulness of service providers and the benefits of seeking treatment from these providers (Jorm et al., 1997a; Angermeyer et al., 1999; Rickwood et al., 2007; Rughani et al., 2011), knowledge and understanding of specific risk factors and causes of mental health problems, and attitudes toward mental illnesses (Jorm et al., 1997a,b). Individuals who held negative views about the effectiveness of mental health services were unlikely to express an intention to access such services (Bayer and Peay, 1997; Angermeyer et al., 1999). Several studies have also shown that people who have sought professional help at some time in their lives have more positive attitudes toward help-seeking than those who have not (Halgin et al., 1987; Lin and Parikh, 1999).

Whilst various attitudinal barriers to help-seeking have been identified, research has also consistently found socio-demographic factors to be associated with positive help-seeking attitudes including female gender (Fischer and Turner, 1970; Yeh, 2002; Vogel and Wester, 2003; Ang et al., 2004; Nam et al., 2010), higher socioeconomic status (Figueroa et al., 1984), and higher educational level (Sheikh and Furnham, 2000; Goh and Ang, 2007). Research has also shown there to be some subcultural factors affecting attitudes toward help-seeking (Fischer and Farina, 1995; Zhang and Dixon, 2003; Goh and Ang, 2007).

In order to better understand attitudes toward help-seeking behavior and related mental health service utilization, various conceptualizations have been adopted and applied. Fischer and Turner (1970) suggested that one's attitude toward receiving help underlies actual help-seeking behavior and this assumption has been the cornerstone of research on help-seeking attitudes. In order to determine attitudes in help-seeking, Fischer and Turner (1970) developed the 29-item Attitudes Toward Seeking Professional Psychological Help (ATSPPH) scale, the most widely used contemporary assessment of help-seeking attitudes. The ATSPPH has consistently shown to have acceptable psychometric properties in a range of samples and the scale has been used extensively in both Western and Eastern settings (Fischer and Farina, 1995; Razali and Najib, 2000; Sheikh and Furnham, 2000; Nam et al., 2010).

This is to our knowledge, the only standardized instrument to access attitudes toward help-seeking that has been both psychometrically examined and used in a sizeable number of studies. Whilst there are a few similar measures, such as the Inventory of Attitudes toward Seeking Mental Health Services (Mackenzie et al., 2004) and the Willingness to Seek Help Questionnaire (Cohen, 1999) these are used far less frequently and have their limitations, including a lack of brevity, inability to assess constructs that are focused on global treatment attitudes, availability of limited psychometric data and they are often not generalizable as they have been validated in specific populations such as students (Kushner and Sher, 1989; Komiya et al., 2000; Mackenzie et al., 2004; Elhai et al., 2008). The authors subsequently developed a shortened uni-dimensional 10-item scale (ATSPPH-SF) which has also been extensively used (Fischer and Farina, 1995). This shortened version similarly has documented psychometric support (Fischer and Farina, 1995; Komiya et al., 2000; Vogel et al., 2005; Elhai et al., 2008).

There have been a few studies that have explored the factor structure of the ATSPPH-SF, with mixed findings. One study among college students and primary care patients found the ATSPPH-SF scale to have a two factor model (Elhai et al., 2008), while a local study conducted in Singapore among trainee teachers and undergraduate teachers found that removing one item and having a 9-item uni-dimensional scale, produced the best fit (Ang et al., 2007). To date, the majority of research using the ATSPPH or ATSPPH-SF scale has focused on specific population sub-groups such as students or teachers, and whilst several studies have looked at Asian populations, the majority of these have been Asians living in Western countries. The gaps in the existing literature warrant the need for multi-ethnic population based research studies which explore ATSPPH. Furthermore, having a greater understanding of attitudes toward help-seeking is imperative as these attitudes have the potential to be mutable, in facilitating individuals' access to treatment (Bhugra and Hicks, 2004).

Singapore is a multi-ethnic country in Southeast Asia, and in 2015, the resident population was 3.9 million, comprising predominantly of Chinese, Malays, and Indians (Department of Statistics, 2015). Ang et al. (2004) explored the effects of gender and sex role orientation on ATSPPH among student trainee teachers in Singapore. They found females had more positive overall attitudes toward professional help-seeking and were more willing to recognize a personal need for professional help compared to males.

Another local study investigated the extent to which people prefer to seek professional help and whether they actually sought professional help for their mental or emotional problems (Ng et al., 2003). Findings revealed that only 37% of the general population would seek professional help if they experienced a serious emotional or mental problem. The authors concluded that while psychiatric disturbance was the most important factor determining mental health service use, attitudes toward seeking professional help were an important enabling factor of utilization and this apparent lack of acceptance contributes to unmet needs. The aims of the current study were to firstly explore the factor structure of the ATSPPH-SF scale among the multi-ethnic general population in Singapore and secondly, determine whether any significant socio-demographic differences exist in terms of help-seeking attitudes.

## Materials and methods

### Participants and procedure

Data from the current study came from a larger comprehensive, population-based, cross-sectional mental health literacy survey conducted between March 2014 and April 2015 among Singapore citizens and Permanent Residents aged 18–65 years, who were residing in Singapore during the survey period. Respondents were randomly selected via a national registry that maintains the names and socio-demographic details such as age, gender, ethnicity, and household addresses of all residents in Singapore. Residents living outside of Singapore, those who were unable to be contacted due to incomplete or incorrect addresses and those who were unable to complete the interview in one of the specified languages were excluded from the survey. Trained interviewers administered the questionnaire in English, Mandarin, Malay, or Tamil, based on the respondent's preference. A total of 3006 people completed the face-to-face interview, equating to an overall response rate of 71.1%.

The study was approved by the relevant institutional and ethics committee (National Healthcare Group Domain Specific Review Board). All respondents provided written informed consent and for those aged below 21 years, written informed consent was also obtained from their legally acceptable representative, parent, or guardian. Additional information pertaining to the methods and procedures is described elsewhere (Subramaniam et al., 2016).

### Survey measures

#### Attitudes toward seeking professional psychological help

The 10-item ATSPPH-SF (Fischer and Farina, 1995) was used to measure general ATSPPH for mental health issues. Items are rated on a 4-point Likert-type scale (3 = Agree, 0 = Disagree), where items 2, 4, 8, 9, and 10 are reverse scored. Scores are then summed together, with higher scores indicating more positive attitudes toward seeking professional help. The correlation between the 10-item short form and the original 29-item scale was 0.87 (Fischer and Farina, 1995).

Socio-demographic information relating to the participants was also collected using a structured questionnaire and included age, gender, ethnicity, marital status, educational status, and income.

#### Cognitive testing and adaptation of ATSPPH-SF scale

Cognitive interviews were conducted with 75 lay members of the population to ensure the items and terminology used in the ATSPPH-SF scale was understood as intended. This cognitive model was applied in a manner that is designed to ultimately improve the quality of survey questions through the study of comprehension, retrieval, judgment, and response processes (Willis, 2004). Respondents were instructed by trained interviewers who systematically probed on whether they could repeat the questions and what came to their mind when they heard a particular phrase or term and they were asked how they decided on their response. Respondents also reported any word they did not understand and any word or expression that they found offensive or unacceptable; and where alternative words or expressions exist for one item or expression, the respondent was asked which of the alternatives conforms better to their usual language. Minor changes were made to the ATSPPH-SF scale, to improve cultural understanding, after seeking permission from the developer (Edward. H. Fischer).

### Statistical analysis

All estimates were weighted to adjust for over sampling and post-stratified for age and ethnicity distributions between the survey sample and the Singapore resident population in the year 2012. Weighted mean and standard error of the mean were calculated for continuous variables, and frequencies and percentages for categorical variables. Confirmatory factor analysis (CFA) and exploratory factor analysis (EFA) were performed to establish the validity of the factor structure of the ATSPPH-SF scale. CFA models were estimated to test the one and two factor structure model proposed previously by Ang et al. (2007) and Elhai et al. (2008), among the whole sample, however this resulted in a poor model fit. We therefore re-analyzed the data using EFA, among a random half of the sample (*n* = 1500), in order to identify the number of underlying factors, with all rotated loadings freely estimated using oblique Geomin rotation method. This was followed by CFA (*n* = 1502) to confirm the factor structure yielded by EFA with the second half of the sample (Neumann et al., 2008). Several criteria were used to determine the number of factors such as eigenvalue-based procedures including the number of eigenvalues >1.0 and scree plot, pattern of loadings on each factor (i.e., number of non-loading or double-loading items), and interpretability of each solution.

All structural equation modeling analyses were performed on polychoric correlation matrixes using Mplus version 7.0 with the weighted least squares with mean and variance adjusted chi-square statistic (WLSMV) estimator for categorical variables. The WLSMV estimation was used due to fact that this estimator is more suited to the ordered-categorical nature of Likert scales than traditional maximum likelihood estimation (Beauducel and Herzberg, 2006).

We used several criteria to determine the best fit model. We chose 0.4 as a cutoff for size of loading to be interpreted (Brown, 2006). Overall model fit was measured using a range of goodness-of-fit statistics based on the following criteria: the comparative fit index (CFI), the Tucker-Lewis index (TLI), the root mean square error of approximation (RMSEA) and standardized root mean square residual (SRMR). Cutoff values suggested by Hu and Bentler (1999) were used—a cutoff value of close to 0.08 for SRMR, close to 0.95 for TLI and CFI, and values smaller than 0.08 or 0.06 for the RMSEA support respectively acceptable and good model fit (Browne and Cudeck, 1993). We calculated the reliability of the scale using Composite Reliability Index (CRI) based on CFA measurement parameters.

$$\begin{array}{l} \rho ={\left(\Sigma \lambda_{\text{i}}\right)}^{2}∕\left[{\left(\Sigma \lambda_{\text{i}}\right)}^{2}+\Sigma \theta_{\text{ii}}\right] \end{array}$$

where ${\left(\Sigma \lambda_{\text{i}}\right)}^{2}$ is the squared sum of unstandardized factor loadings, and Σθ_ii_ is the sum of unstandardized measurement error variances (Raykov, 1997, 2004; Brown, 2006).

We also conducted separate multivariable linear regressions to examine factors associated with each of the ATSPPH-SF scores (dependent variables) to examine which of the following dummy coded variables (independent variables) predicted the ATSPPH-SF scores: age, gender, ethnicity, marital status, education, employment status, and income.

## Results

Table 1 shows the socio-demographic characteristics of the sample (*n* = 3006). The mean age of the respondents was 40.9 years. About 50.9% of the respondents were males, 74.7% were Chinese, 12.8% were Malays, 9.1% were Indians, and 3.3% belonged to other ethnic groups.

**Table 1:** **Socio-demographic characteristics of the study sample**.

	**N**	**Weighted %**	**SE**
**AGE GROUP**			
18–34 years	1152	34.4	0.04
35–49 years	896	35.2	0.04
50–65 years	958	30.4	0.1
**GENDER**			
Female	1506	49.1	1.3
Male	1500	50.9	1.3
**ETHNICITY**			
Chinese	1034	74.7	0.04
Malay	977	12.8	0.01
Indian	963	9.1	0.01
Others	32	3.3	0.04
**MARITAL STATUS**			
Married	1916	64.0	1.0
Never married	927	31.4	0.9
Others (Divorced, Widowed, Separated)	162	4.6	0.5
**EDUCATION**			
Primary and below	431	13.3	0.8
Secondary education include O/N level	820	25.8	1.0
A level, polytechnic and other diploma	999	31.3	1.1
University	756	29.6	1.1
**EMPLOYMENT STATUS**			
Employed	2227	77.6	1.0
Housewife/homemaker	378	8.7	0.6
Retired	78	3.0	0.4
Student	203	6.7	0.5
Unemployed	120	3.9	0.5
**INCOME**			
<$SGD2000	1346	40.5	1.2
$SGD2000-5999	1162	46.4	1.3
$SGD6000 and above	294	13.1	0.9

Table 2 provides the factor loadings and model fits for the CFA and EFA models of the ATSPPH-SF scale. First we examined a two factor structure for the ATSPPH-SF as proposed by Elhai et al. (2008) using CFA (Model 1). Although this model indicated higher factor loadings (all loadings above 0.4), the fit indices were poor especially for CFI and TLI indices [$\chi_{\left(df\right)}^{2}$ = 252.427(25), CFI = 0.879, TLI = 0.884, RMSEA = 0.055, SRMR = 0.047]. We also examined the nine item uni-dimensional model suggested by Ang et al. (2007) however found that this had poor factor loadings and fit indices (Model 2). Therefore, we decided to further analyse using EFA. In EFA, a three factor structure provided a good fit, $\chi_{\left(df\right)}^{2}=$ 33.64(15), CFI = 0.978, TLI = 0.966, RMSEA = 0.029, SRMR = 0.028, with high factor loadings (Model 3). Inspection of eigenvalues >1.0 and scree plot supported the three factor solution. The findings from the factor analysis revealed that the ATSPPH-SF scale formed three distinct dimensions comprising “Openness to seeking professional help,” “Value in seeking professional help,” and “Preference to cope on one's own.” Following this, we then used CFA to confirm this new structure (Model 4). The CRI for the overall scale as well as for the “Openness to seeking professional help,” “Value in seeking professional help,” and “Preference to cope on one's own” dimensions were 0.97, 0.88, 0.88, and 0.86, respectively.

**Table 2:** **Factor loadings and model fits for the CFA and EFA models of the 10-item Attitudes Toward Seeking Professional Psychological Help Scale**.

Item^*^		**CFA (Overall sample = 3002)**			**EFA (Subsample1 = 1500)**			**CFA (Subsample2 = 1502)**		
		**Model 1**		**Model 2**	**Model 3**			**Model 4**		
		**f1**	**f2**	**f1**	**f1**	**f2**	**f3**	**f1**	**f2**	**f3**
ATSPPH1		**0.609**	**0.450**	**0.514**	0.126	**0.461**	0.036		**0.692**	
ATSPPH2				0.398	**0.514**	−0.115	0.290	**0.387**		
ATSPPH3		**0.573**		**0.505**	**0.562**	0.253	−0.295	**0.791**		
ATSPPH4			**0.476**	0.351	−0.014	0.092	**0.554**			**0.501**
ATSPPH5		**0.826**		**0.752**	0.229	**0.647**	0.064		**0.846**	
ATSPPH6		**0.606**		**0.520**	−0.027	**0.630**	0.107		**0.632**	
ATSPPH7		**0.614**			0.228	**0.534**	−0.089		**0.582**	
ATSPPH8			**0.487**	**0.471**	**0.504**	−0.018	0.199	**0.504**		
ATSPPH9			**0.612**	**0.442**	0.068	0.029	**0.723**			**0.574**
ATSPPH10			**0.688**	**0.517**	0.070	0.059	**0.596**			**0.889**
**CORRELATION AMONG THE FACTORS**										
	f1 with f2	0.359			0.394			0.734		
	f1 with f3				0.047			0.297		
	f2 with f3				0.191			0.237		
**RECOMMENDATION FOR PREFERRED INDICES**										
Chi-Square		252.427		653.026	33.640			107.841		
df		25		20	15			22		
CFI	>0.95	0.879		0.605	0.978			0.913		
TLI	>0.95	0.884		0.586	0.966			0.913		
RMSEA	< 0.08	0.055		0.103	0.029			0.051		
SRMR	< 0.08	0.047		0.124	0.028			0.058		

* *Item wording is listed under Appendix 1*.

*Model 1, Elhai et al., 2008*.

*Model 2, Ang et al., 2007*.

*Bold indicates factor loading values greater than 0.4 within the expected dimensions*.

Table 3 shows the socio-demographic correlates of ATSPPH-SF factor scores calculated by summing items with substantial loadings (>0.30) derived from the EFA Model 3. Multiple linear regression analyses revealed that age, ethnicity, marital status, education, and income were significantly associated with the ATSPPH-SF factors. Those aged 18–34 years and never married, were significantly associated with higher “Openness to seeking professional help,” while Malay ethnicity and lower education were significantly associated with lower openness scores. Both Malay and Indian ethnicity, secondary/O and N level education and A level/diploma, were significantly associated with lower “Value in seeking professional help” scores, while lower education and having an income of $SGD2000-5999 were significantly associated with higher “Preference to cope on one's own” scores.

**Table 3:** **Socio-demographic correlates of Attitudes Toward Seeking Professional Psychological Help**.

	**Openness to seeking professional help**				**Value in seeking professional help**				**Preference to cope on one's own**			
	**β**	**95% CI**		***P-value***	**β**	**95% CI**		***P-value***	**β**	**95% CI**		***P-value***
**AGE GROUP**												
18–34 years	0.831	0.404	1.259	<**0.0001**	0.128	−0.128	0.384	0.328	−0.105	−0.511	0.301	0.612
35–49 years	0.144	−0.231	0.519	0.451	0.056	−0.185	0.297	0.65	−0.004	−0.368	0.361	0.984
50–65 years	1.000				1.000				1.000			
**GENDER**												
Female	−0.149	−0.418	0.12	0.278	0.139	−0.038	0.315	0.123	0.004	−0.272	0.279	0.98
Male	1.000				1.000				1.000			
**ETHNICITY**												
Chinese	1.000				1.000				1.000			
Malay	−0.309	−0.548	−0.07	**0.011**	−0.386	−0.544	−0.228	<**0.0001**	−0.084	−0.316	0.147	0.474
Indian	−0.133	−0.371	0.105	0.275	−0.307	−0.466	−0.148	**0.0002**	−0.002	−0.236	0.233	0.989
Others	0.049	−0.941	1.039	0.923	−0.163	−0.956	0.629	0.686	0.144	−1.037	1.325	0.811
**MARITAL STATUS**												
Married	1.000				1.000				1.000			
Never married	0.514	0.175	0.852	**0.003**	0.03	−0.181	0.241	0.78	0.296	−0.05	0.643	0.093
Others (Divorced, Widowed, Separated)	−0.161	−0.694	0.371	0.553	0.046	−0.355	0.448	0.822	−0.175	−0.742	0.392	0.546
**EDUCATION**												
Primary and below	−0.954	−1.547	−0.361	**0.002**	−0.197	−0.552	0.157	0.275	1.406	0.875	1.936	<**0.0001**
Secondary education include O/N level	−0.486	−0.922	−0.051	**0.029**	−0.422	−0.687	−0.157	**0.002**	1.206	0.775	1.637	<**0.0001**
A level, polytechnic and other diploma	−0.027	−0.385	0.332	0.884	−0.267	−0.496	−0.038	**0.022**	0.815	0.463	1.167	<**0.0001**
University	1.000				1.000				1.000			
**EMPLOYMENT STATUS**												
Employed	1.000				1.000				1.000			
Housewife/homemaker	0.26	−0.222	0.742	0.29	0.058	−0.287	0.402	0.743	−0.164	−0.667	0.338	0.521
Retired	−0.008	−0.717	0.701	0.983	0.498	−0.027	1.022	0.063	0.146	−0.471	0.762	0.643
Student	0.396	−0.141	0.933	0.148	0.127	−0.2	0.453	0.447	−0.482	−1.011	0.046	0.074
Unemployed	0.019	−0.677	0.716	0.957	0.027	−0.346	0.399	0.888	0.093	−0.647	0.833	0.805
**INCOME**												
>$SGD6000	1.000				1.000				1.000			
<$SGD2000	−0.461	−0.997	0.076	0.092	−0.23	−0.568	0.109	0.184	0.472	−0.05	0.994	0.077
$SGD2000-5999	−0.269	−0.718	0.18	0.24	−0.106	−0.399	0.188	0.48	0.602	0.159	1.046	**0.008**

*Bold indicates significant associations at p value < 0.05*.

## Discussion

This study examined the factor structure of the ATSPPH-SF scale and determined the socio-demographic differences relating to ATSPPH. EFA revealed that the ATSPPH-SF scale comprised three distinct components; the first relates to openness to seeking professional help for psychological or emotional problems, the second is about the value in seeking professional help, while the third relates to coping on one's own and choosing not to seek psychological help. It was evident that EFA supports this three factor structure given the good fit, high factor loading as well a good reliability. CFA also confirmed an acceptable model fit with CFI and TLI cut offs being above 0.95 (Bentler, 1990), while the RMSEA cut off was below 0.8. Further research to reconfirm this factor structure in an external dataset is warranted.

This is somewhat different to previous research which has found two distinct factors comprising “Openness to Seeking Treatment for Emotional Problems,” and “Value and Need in Seeking Treatment” (Elhai et al., 2008) among a college student and medical patient population in the USA. It is also different from that associated with the original ATSPPH-SF which had a one factor structure (Fischer and Farina, 1995). In a study among trainee teachers and undergraduate students in Singapore, Ang et al. (2007) also tested the uni-dimensional factor structure of the ATSPPH-SF scale using CFA. In both population subgroups they found item 7, “A person with an emotional problem is not likely to solve it alone; he or she is more likely to solve it with professional help” to be problematic, due to the double-barreled nature of the question. Due to the problematic wording and poor factor loading, the item was dropped and CFA revealed a very good fit for a uni-dimensional, 9-item scale; however when this model was applied to the general Singapore population used in our study, the factor loading and fit indices were poor. The small sample size used by Ang et al. (2007), which was specific to student and trainee teacher populations could explain the differences in factor structures between this and our study, which used a large generalizable, multi-ethnic sample.

Among the three factor ATSPPH-SF scale, we found various socio-demographic correlates associated with each factor. Firstly younger age (18–34 years) was significantly associated with increased openness to seek professional psychological help. The research to date has shown that age related differences in help-seeking attitudes are inconsistent. Older adults have been known to display negative attitudes to help-seeking (Estes, 1995; Segal et al., 2005) and whilst there can be multiple barriers to seeking professional help, actual attitudes of older adults are seen to be the most significant (Currin et al., 1998; Hatfield, 1999). Contrary to this, other studies have shown older adults' attitudes toward seeking help are generally positive (Robb et al., 2003). Given these inconsistencies, there is a need to further explore the impact of age on openness to seeking psychological help and help-seeking attitudes in general.

Interestingly we found there to be no gender differences in relation to any of the three ATSPPH-SF factors. The extant literature has shown that females are significantly more likely to have positive attitudes toward seeking professional psychological help, compared to their male counterparts (Fischer and Turner, 1970; Yeh, 2002; Addis and Mahalik, 2003; Vogel and Wester, 2003; Ang et al., 2004; Nam et al., 2010). There is however some evidence which supports the lack of gender differences with regards to ATSPPH, which has largely come from studies among Asian Americans and Asian international students (Atkinson et al., 1995; Zhang and Dixon, 2003) which may suggest that ethnic or cultural differences may intersect with gender differences and influence help-seeking attitudes.

The literature has shown that various ethnic groups differ widely in relation to help-seeking patterns, utilization and attitudes toward mental health services (Bayer and Peay, 1997; Van OS et al., 1997) and therefore it was not surprising to find ethnic differences in relation to ATSPPH in our study. Malays were significantly less likely to be open to seeking psychological help, whilst both Malays and Indians were less likely to value seeking psychological help. These findings can be explained by various influencing factors.

The first relates to religion. A study conducted in Malaysia examining the impact of culture on illness perceptions and help-seeking behaviors among Chinese and Malays found cultural differences, whereby Malays endorsed religious attributions and help-seeking behaviors such as prayer and seeking traditional treatment from “*bomohs*” (traditional healers), more than Chinese (Edman and Koon, 2000). These findings are also similar to those of Hatfield et al. (1996) which found the value of Islamic prayer to be an important way of seeking help for mental illness and could therefore explain the ethnic differences observed in our study. The second is in relation to illness attribution. Help-seeking attitudes are often linked to illness attributions, where mental illnesses are often attributed to supernatural causes by Indians and Malays (Razali et al., 1996; Banerjee and Roy, 1998; Sheikh and Furnham, 2000) which may influence their help-seeking attitudes. Finally, the third is in relation to cultural and family influences. Research has consistently shown that Asians prefer to seek help from less formal sources such as family for mental health problems (Lin et al., 1982; Leong, 1986; Atkinson et al., 1995; Yeh, 2002). Alternatively, it could be the family that decides where further help should be sought (Lin and Cheung, 1999; Razali and Najib, 2000); these preferences and influences are likely to explain why Malays and Indians see less value in seeking professional psychological help.

Stigma associated with seeking psychological help could also be an underlying factor. In a recent study among the same population, which examined the extent and correlates of stigma toward people with mental illness, the authors found that those of Malay and Indian ethnicity were significantly more likely to perceive those with a mental illness to be “weak not sick” (Subramaniam et al., 2016). Given that people with a mental illness were characterized as having a weakness, under the control of the person, rather than a real medical problem, this may affect their openness to and value in seeking psychological help.

Marital status has been shown to be a predictor of ATSPPH and in our study we found that those who were never married were more open to seeking professional help. Difficulties forming or maintaining relationships and lack of social support from a partner may be strong impetuses for those not married to be more open to seeking help (Leaf et al., 1988; Gallo et al., 1995). Marital status could also be related to age, whereby younger people are less likely to be married and were also found to be more open to seeking professional help.

Lower education has consistently been associated with negative ATSPPH (Sheikh and Furnham, 2000; Al-Krenawi et al., 2004; Goh and Ang, 2007), a finding which is consistent with our study where we found lower education was associated with less openness to and value in seeking psychological professional help. Our findings suggest that respondents with higher education viewed psychological help-seeking more favorably, which can be explained by having greater knowledge of psychological help-seeking options and/or the associated benefits of such treatments. Interestingly, lower education was also significantly associated with increased self-coping or a greater preference to cope on one's own and again this may be due to limited knowledge or understanding about the benefits of seeking professional psychological help.

The findings from our study should be viewed in light of the following limitations. Whilst we looked at various socio-demographic predictors of ATSPPH, we did not explore other characteristics such as prior contact with or exposure to mental health services. Whilst the differences in attitudes were determined using multiple logistic regression, further examination or alternative analyses such as multi-group CFA or latent mean differences are recommended in the future. Furthermore, as the current study focused on attitudes in relation to seeking professional psychological help, there is a need for future research to examine whether attitudes are indeed associated with actual utilization of and satisfaction with services. Finally, the reliance on self-report by the respondents has the possibility of social desirability bias, especially since the questions measured ATSPPH.

### Implications and conclusion

This study, among a nationally representative sample, with a response rate of 71%, has indicated various socio-demographic correlates related to ATSPPH including age, ethnicity, marital status, and education. Given that there is a tremendous treatment gap associated with mental illnesses in Singapore (Chong et al., 2012a), there is a need to further investigate the associations between help-seeking attitudes and actual help-seeking behavior. Research exploring the effects of culture, religion, and ethnicity on help-seeking attitudes to gain a greater understanding of how these complex and inter-related constructs influence and impact help-seeking attitudes, is also warranted. As ethnicity was a significant correlate of help-seeking attitudes, where Malays and Indians were less open to seeking professional help and saw less value in such help, culturally appropriate education efforts to highlight and inform these population sub-groups about professional psychological and the benefits of such treatment, are required. These findings also have important implications in terms of service planning and increasing utilization; given that those with less education had a greater preference to cope on one's own, there is a need to improve outreach and encourage help-seeking from professional psychological services, via tailored and supportive interventions.

In view of the detrimental effects associated with under-utilization, treatment delays, and gaps in mental healthcare delivery, there is a need to address and gain a greater understanding about help-seeking attitudes, behaviors, and preferences for people with a mental illness. Researchers have sought to explain underutilization of professional psychological services among Asian populations and three major reasons have been postulated: (1) lack of trust in helping professionals and their services (Nu, 1987; Pan, 1996); (2) lack of knowledge about the availability of services (Leong, 1986); and the stigma associated with formal help-seeking (Leong, 1986; Mau and Jepsen, 1988). Exploring these potential barriers within the Singapore context could help to improve and change help-seeking attitudes and behaviors in the future.

## Author contributions

LP was involved with the study design, collected, and verified data and wrote the manuscript. EA was involved in the data analysis and interpretation and provided inputs into the manuscript. SAC assisted in study design, interpreted the data, and provided intellectual inputs on the manuscript. SP, SS, BYC, JV played an active role in data collection, clean up, refining analysis plan, and drafting the manuscript. LPO provided clinical inputs and interpretations into the findings and provided inputs and edits to the manuscript. JT was involved with the study design and provided inputs into the manuscript. MS supervised the overall study design, provided inputs on the manuscript content, and approved the manuscript version to be published.

### Conflict of interest statement

The authors declare that the research was conducted in the absence of any commercial or financial relationships that could be construed as a potential conflict of interest.

## Appendix 1. Revised ATSPPH-SF items

1. If I thought I was having a mental breakdown, my first thought would be to get professional attention.
2. Talking about problems with a psychologist seems to me as a poor way to get rid of emotional problems.
3. If I were experiencing a serious emotional crisis, I would be sure that psychotherapy would be useful.
4. I admire people who are willing to cope with their problems and fears without seeking professional help.
5. I would want to get psychological help if I were worried or upset for a long period of time.
6. I might want to have psychological counseling in the future.
7. A person with an emotional problem is not likely to solve it alone; he or she is more likely to solve it with professional help.
8. Given the amount of time and money involved in psychotherapy, I am not sure that it would benefit someone like me.
9. People should solve their own problems, therefore, getting psychological counseling would be their last resort.
10. Personal and emotional troubles, like most things in life, tend to work out by themselves.
